# Impact of alternative terminology for depression on help‐seeking intention: A randomized online trial

**DOI:** 10.1002/jclp.23410

**Published:** 2022-07-08

**Authors:** Jenna Smith, Erin Cvejic, Tara J. Lal, Alana Fisher, Marguerite Tracy, Kirsten J. McCaffery

**Affiliations:** ^1^ Sydney Health Literacy Lab, Sydney School of Public Health, Faculty of Medicine and Health The University of Sydney Sydney New South Wales Australia; ^2^ University of New England Armidale New South Wales Australia; ^3^ eCentre Clinic, School of Psychological Sciences Macquarie University Sydney New South Wales Australia

**Keywords:** depression, burnout, disease‐labeling, stigma, help‐seeking, communication

## Abstract

**Objective:**

People with depression experience barriers to seeking professional help. Different diagnostic terminology can influence people's treatment/management preferences. The aim of this study was to investigate how alternative depression diagnostic labels and recommendations impact help‐seeking intentions and psychosocial outcomes.

**Methods:**

Participants (18–70 years) were recruited using an online panel (Australia) to complete a randomized controlled trial. They read a hypothetical scenario where they discussed experiencing depressive symptoms with their GP and were randomized to receive one of four diagnoses (“depression,” “burnout,” “functional impairment syndrome” [fictitious label], no label [control]), and one of two follow‐up recommendations (“clinical psychologist,” “mind coach”). Primary outcome: help‐seeking intention (5‐point scale, higher = greater intention); secondary outcomes: intention to speak to boss, self‐stigma, worry, perceived severity, illness perceptions, and personal stigma.

**Results:**

A total of 676 participants completed the survey. There was no main effect of diagnostic label on help‐seeking intention or stigma outcomes. Intention to speak to a boss was higher with the depression compared to burnout label (MD = 0.40, 95% CI: 0.14–0.66) and perceived severity was higher with the depression label compared to control (MD = 0.48, 95% CI: 0.22–0.74) and all other labels. Those who received the “clinical psychologist” recommendation reported higher help‐seeking intention (MD = 0.43, 95% CI: 0.25–0.60) and treatment control (MD = 0.69, 95% CI: 0.29–1.10) compared to the “mind coach” recommendation.

**Conclusion:**

Findings highlight the success of efforts to promote help‐seeking from clinical psychologists for depression. If burnout is considered a separate diagnostic entity to depression, greater awareness around what such a diagnosis means may be needed. Future research should examine how different terminologies surrounding other mental health conditions impact help‐seeking and stigma.

## 1 INTRODUCTION

Many people with depression are reluctant to seek professional help, for reasons including perceived stigma and self‐stigma (Clement et al., 2015). Perceived stigma is the belief that other people hold negative attitudes toward them and self‐stigma occurs when an individual then directs this attitude onto themselves (Corrigan & Watson, 2002). Previous research has shown that strategies employed to reduce stigmatizing beliefs of depression (e.g., educating the public about the genetic and neurobiological nature of mental disorders) have failed to translate into more tolerant attitudes toward people with a mental illness (Schomerus et al., 2012). Such strategies can increase feelings of pessimism and helplessness due to deterministic beliefs (Haslam & Kvaale, 2015), and remove the depressed person's individual agency to seek help and benefit from behavioral and psychosocial interventions (Lebowitz, 2014). However, the stigma associated with a diagnosis of depression in more recent times is unknown.

To mitigate the perceived and self‐stigmatizing beliefs that a mental health diagnosis such as depression might elicit, individuals tend to avoid seeking professional help and thus avoid the stigmatizing label of depression altogether (Corrigan et al., 2014). Research has indicated that, when developing interventions to reduce stigma and enable help‐seeking, it may be particularly important to address the labeling associated with depression, and referring to it as a “health condition” instead of “mental illness” may be helpful (Barney et al., 2009). However, the effect of the depression label itself is complex considering the great flexibility and heterogeneity in the use of the term to indicate both normal and psycho‐medical/clinical distress and impaired functioning (Bröer & Besseling, 2017). For individuals who indicate mild symptoms within the subclinical range for depression and which do not meet diagnostic criteria for major depressive disorder (MDD), the impact of receiving a depression label on help‐seeking intentions and stigma is unknown. More specifically, it is unknown whether using the label “depression” in such cases is beneficial, or whether it may be less stigmatizing to avoid using “depression” or even use alternative terminology to describe such symptoms.

Experiences of psychological distress or depressive symptoms in the workplace is one context in which the impacts of depression labeling could be examined, as there is continued debate surrounding the relationship between burnout and depressive symptoms. Someone experiencing burnout is more susceptible to developing depression and vice‐versa (Bianchi et al., 2015). A recent meta‐review described moderate evidence for work‐related factors such as high job demands, role stress, and low social support as being associated with a greater risk of developing mental health issues (Harvey et al., 2017). The definitive nature of these associations is yet to be established empirically, but significant overlap exists between burnout and MDD in terms of symptoms and clinical features (Bianchi et al., 2015). As of 2019, burnout has been included in the International Classification of Diseases as an occupational phenomenon, not a medical condition, that results from chronic workplace stress, and is characterized by exhaustion, cynicism toward or distance from one's job and reduced professional efficacy (World Health Organization, 2019). In medical settings, the burnout label could be less stigmatizing than providing a depression diagnosis for mild symptoms, and may therefore translate to greater help‐seeking in the workplace context. One French study found that participants reported less stigma associated with a burnout label compared to a depression label, but no differences in help‐seeking intentions (Bianchi et al., 2016). However, only school teachers were recruited for this study and although it was a large study, participants were not randomized to each version of the survey that assessed responses to the different labels (Bianchi et al., 2016). This design has a high risk of bias and therefore we can only have limited confidence in its findings. Considering the important role of labeling in the process of stigma‐related beliefs, the complex and heterogeneous nature of the term depression and its relationship to burnout and workplace stress, examining the effect of these labels on beliefs, attitudes, and help‐seeking intentions is warranted. It is unknown whether communicating a depression diagnosis in cases in which psychological distress is experienced in a workplace setting may alleviate the negative effects of stigma and enable greater help‐seeking or whether it may be more worthwhile to delay such a diagnosis or use alternative terminology.

The primary aim of this study was to investigate the effects of a “depression,” “burnout” and a novel fictitious diagnostic label (“functional impairment syndrome”) which has no pre‐existing history or culture of stigma, compared to no diagnosis (control), on hypothetical help‐seeking intentions and psychosocial outcomes by presenting workplace stress and mental illness scenarios. This study also aimed to examine the impact of (i) the different diagnostic labels on reports of self‐stigma, personal stigma (attitudes toward diagnosis), perceived severity, and cognitive illness perceptions; and (ii) different labels used to describe the mental healthcare provider (clinical psychologist vs. mind coach) on help‐seeking intentions and psychosocial outcomes.

## 2 MATERIALS AND METHODS

### 2.1 Participants

Participants were aged 18–70 years, living in Australia, recruited using a social research company specializing in online panel survey research (Dynata). This company maintains an extensive database of over 100,000 Australians (whose demographics strongly align with the national population), who have indicated their willingness to be involved in online survey research. Eligibility criteria were kept deliberately broad to include having an adequate understanding of English and currently living in Australia.

### 2.2 Design

This online study used a randomized parallel 4 × 2 design. Participants were randomized to one of eight hypothetical scenarios, where diagnostic label (no label vs. “depression” vs. “burnout” vs. “functional impairment syndrome”; allocation ratio 1:1:1:1) and recommendation label (“clinical psychologist” vs. “mind coach”; allocation ratio 1:1) were varied. This design allowed for causal inference regarding the effects of the diagnostic labels and recommendation labels, and their interactions.

### 2.3 Intervention

#### 2.3.1 Hypothetical scenario of a doctor's visit

Participants were asked to imagine that over the past few months they had not been feeling themselves, so they went to their general practitioner (GP) and explained how they had been feeling. Each scenario depicted an appointment with the participant's GP. The symptoms described were the same in all conditions, except for a slight difference between men and women, whereby women felt “upset” and men felt “irritable,” as irritability has been identified as an important depressive symptom reported by men (Martin et al., 2013). See Box 1 for the exact wording of male and female versions of the scenario, as well as Supporting Information for the wording of diagnostic labels and recommendation labels provided by the GP.

Box 1. **Hypothetical scenario**

*You have not been feeling yourself for a few weeks now, so you go to your general practitioner and explain how you've been feeling*:

***FEMALE***:

**Your GP asks, “What can I help you with today?”**

You say you just don't feel like your usual self. Over the last couple of months, you've been finding work really exhausting. Most nights you are not sleeping as well as usual and you feel tired a lot of the time. Nowadays you also find yourself needing a drink (or three!) just to “switch off” from all the workday stress.

**The GP says, “Tell me more about how your work is going and how you're feeling.”**

You say that lately, you're struggling to make it to work on time and finish your usual tasks. You've also been taking more sick days than usual. You're normally quite sociable at work, but you haven't really felt like being around people and just feel like you want to stay at home. When you are at work, you don't really speak to anyone about anything except work matters.

You say you usually enjoy your job and feel good at it, but now you can't help feeling negative about it and like you don't have much to offer anyone. Your boss noticed a change and arranged a meeting with you. During the meeting you find yourself becoming very upset and emotional, which is unusual for you.

***MALE***:

**Your GP asks, “What can I help you with today?”**

You say you just don't feel like your usual self. Over the last couple of months, you've been finding work really exhausting. Most nights you are not sleeping as well as usual and you feel tired a lot of the time. Nowadays you also find yourself needing a drink (or three!) just to “switch off” from all the workday stress.

**The GP says, “Tell me more about how your work is going and how you're feeling.”**

You say that lately, you're struggling to make it to work on time and finish your usual tasks. You've also been taking more sick days than usual. You're normally quite sociable at work, but you haven't really felt like being around people and just feel like you want to stay at home. When you are at work, you don't really speak to anyone about anything except work matters.

You say you usually enjoy your job and feel good at it, but now you can't help feeling negative about it and like you don't have much to offer anyone. Your boss noticed a change and arranged a meeting with you. During the meeting you found yourself getting irritable, which is unusual for you.

#### 2.3.2 Diagnostic label intervention

Participants were randomized to one of four diagnostic label groups. The first group received a hypothetical scenario in which the doctor provided no diagnostic label for their reported symptoms (i.e., control group). For the second group, the doctor provided the diagnosis of “depression” to explain their symptoms. The third group received the diagnosis of “burnout” for their reported symptoms and the final group received the fictional diagnostic label of “functional impairment syndrome.”

#### 2.3.3 Recommendation label intervention

Participants were also randomized to receive one of two recommendations for follow‐up help from a mental healthcare provider; either to see a “clinical psychologist” or a “mind coach.” This term was chosen as it was a non‐branded term which was previously used in a scalable mental health coaching research program that aimed to boost resilience and enhance mental wellbeing to reduce risk of mental health issues in first responders (Joyce et al., 2019).

### 2.4 Procedure

The study was conducted online using Qualtrics software. Interested participants read the Participant Information Sheet and Consent Form and were told that the study was about occupational stress. Those who consented to participate were assessed for eligibility. Eligible and consenting participants were then randomized to one of eight hypothetical scenarios. The survey software randomized participants using the Mersenne Twister pseudorandom number generator, which creates allocation sequences. Participants and researchers were blinded to the allocation sequence until data collection was complete. Participants then completed the outcome measures, followed by questions regarding demographic and health characteristics (age, gender, relationship status, education, ethnicity, occupation, history of mental illness, and current depression symptoms via the patient health questionnaire‐9 [PHQ‐9]). Pop‐up text bubbles were available throughout the survey to remind participants of the scenario when completing outcome measures.

### 2.5 Outcomes

A number of validated and adapted measures were used to assess primary and secondary outcomes (see Table 1) immediately after interventions. For the primary outcome (help‐seeking intention), participants were asked to explain their response in a free‐text box. Intention to speak to boss was included given that key management strategies for burnout likely involve addressing work stressors and periods of sick leave (Parker & Tavella, 2021).

**Table 1:** Outcome measures

Outcome	Measure
Help‐seeking intention (Copp et al., 2017; Fisher et al., 2012	Single item: “If you were in this scenario, how likely would you be to seek help from a *clinical psychologist/mind coach*?” 5‐point scale (1 = very unlikely to 5 = likely)
Intention to speak to boss	Single item: “If you were in this scenario, how likely would you be to speak to your boss about what the GP has told you?” 5‐point scale (1 = very unlikely to 5 = very likely)
Self‐stigma (Barney et al., 2010)	16 items (4 subscales), 5‐point scale (1 = strongly disagree to 5 = strongly agree)
* Shame*	Four items: “I would feel ashamed,” “I would feel embarrassed,” “I would feel inferior to other people,” “I would feel disappointed in myself”
* Selfblame*	Four items: “I would think I should be able to cope with things,” “I would think I should be able to pull myself together,” “I would think I should be stronger,” “I would think I only had myself to blame”
* Help‐seeking inhibition*	Four items: “I would feel embarrassed about seeking professional help,” “I would feel embarrassed if others knew I was seeking professional help,” “I would see myself as weak if I took medication,” “I wouldn't want people to know that I wasn't coping”
* Social inadequacy*	Four items: “I would feel I couldn't contribute much socially,” “I would feel inadequate around other people,” “I would feel like I was good company”^a^, “I would feel like a burden to other people”
Worry (Scherer et al., 2013)	Single item: “How worried would you be about your *situation/depression/burnout/functional impairment syndrome?*” 7‐point scale (1 = not at all to 7 = extremely)
Perceived severity (Courneya et al., 2001; Fisher et al., 2012)	Single item: “On a scale of 1–7, please rate the degree to which you agree or disagree with the following statement: I feel that *being in this situation/having depression/having burnout/having functional impairment syndrome* would be serious.” 7‐point scale (1 = strongly disagree to 7 = strongly agree)
Illness perceptions (Broadbent et al., 2006)	“Imagining you were in this situation…”
	0–10 response scale (higher scores=more threatening view of the illness)
* Consequences*	“How much would your *situation/depression/burnout/functional impairment syndrome* affect your life?”
* Timeline*	“How long do you think your *situation/depression/burnout/functional impairment syndrome* would continue?”
* Personal control*	“How much control do you feel you would have over your *situation/depression/burnout/functional impairment syndrome*?”
* Treatment control*	“How much do you think treatment could help your *situation/depression/burnout/functional impairment syndrome*?”
Personal stigma (Griffiths et al., 2004) (respondents own attitudes toward diagnosis)	Nine items, 5‐point scale (0 = strongly disagree to 4 = strongly agree), score range: 0–36
	“People *in this situation/with depression/with burnout/with functional impairment syndrome* could snap out of it if they wanted.”
	“Having *this experience/depression/burnout/functional impairment syndrome* is a sign of personal weakness.”
	“*This experience/Depression/Burnout/Functional impairment syndrome* is not a real medical illness.”
	“People *in this situation/with depression/with burnout/with functional impairment syndrome* are dangerous.”
	“It is best to avoid people *in this situation/with depression/with burnout/with functional impairment syndrome*, so you don't start feeling like this yourself.”
	“People *in this situation/with depression/with burnout/with functional impairment syndrome* are unpredictable.”
	“If I had *this experience/depression/burnout/functional impairment syndrome* I would not tell anyone.”
	“I would not employ someone if I knew they had been *in this situation/diagnosed with depression/diagnosed with burnout/diagnosed with functional impairment syndrome*.”
	“I would not vote for a politician if I knew they had been *in this situation/diagnosed with depression/diagnosed with burnout/diagnosed with functional impairment syndrome*.”

*Note*: Wording for each outcome measure was adapted for relevance for present study. Sources of original development and validation for each measure are cited.

^a^ Reverse scores.

### 2.6 Sample size

Given the absence of prior randomized studies sufficiently similar to the proposed study to inform anticipated effect sizes for our primary outcome, a pilot study was conducted with 160 participants (i.e., 20 participants randomized to each condition). The observed parameter estimates from the model of the primary outcome (help‐seeking intention) were used to inform the subsequent sample size calculation (by simulation). To have at least 80% power to detect statistically significant main effects (at *p* < 0.05) of diagnosis (corresponding to a Cohen's *f* effect size of approximately 0.15) for the primary outcome, it was estimated that 80 participants would be needed per condition (i.e., at least 640 in total). A sample of this size would also provide >90% power to detect small main effects (Cohen's *f*: 0.14) of recommendation. We aimed to over sample to account for dropouts and invalid responses. Pilot study data was not included in the final responses included in analysis.

### 2.7 Data analysis

Statistical analyses were conducted in Stata/IC v 16.1 (StataCorp). Overall differences between randomized conditions were analyzed using linear regression models. All statistical models included diagnostic label (no label, depression, burnout, functional impairment syndrome) and recommendation label (clinical psychologist, mind coach) as categorical covariates, as well as their interaction, whilst controlling for gender (to account for the minor differences in the scenarios presented to male and female participants). Results are presented for main effects (the effect of the study factor averaged across all other variables in the model) for label and recommendation, as well as their interaction. Planned simple contrasts were also conducted to test any differences between the different diagnostic labels (employing a Bonferroni‐adjusted significance threshold of *p* = 0.008 for multiple pairwise comparisons). The study statistician was blinded to the allocation groups until completion of the primary analysis. Exploratory analyses were also conducted by age group, gender, education, mental health history and depression history to examine any interactions by diagnostic label or by recommendation label.

## 3 RESULTS

**Figure 1:**
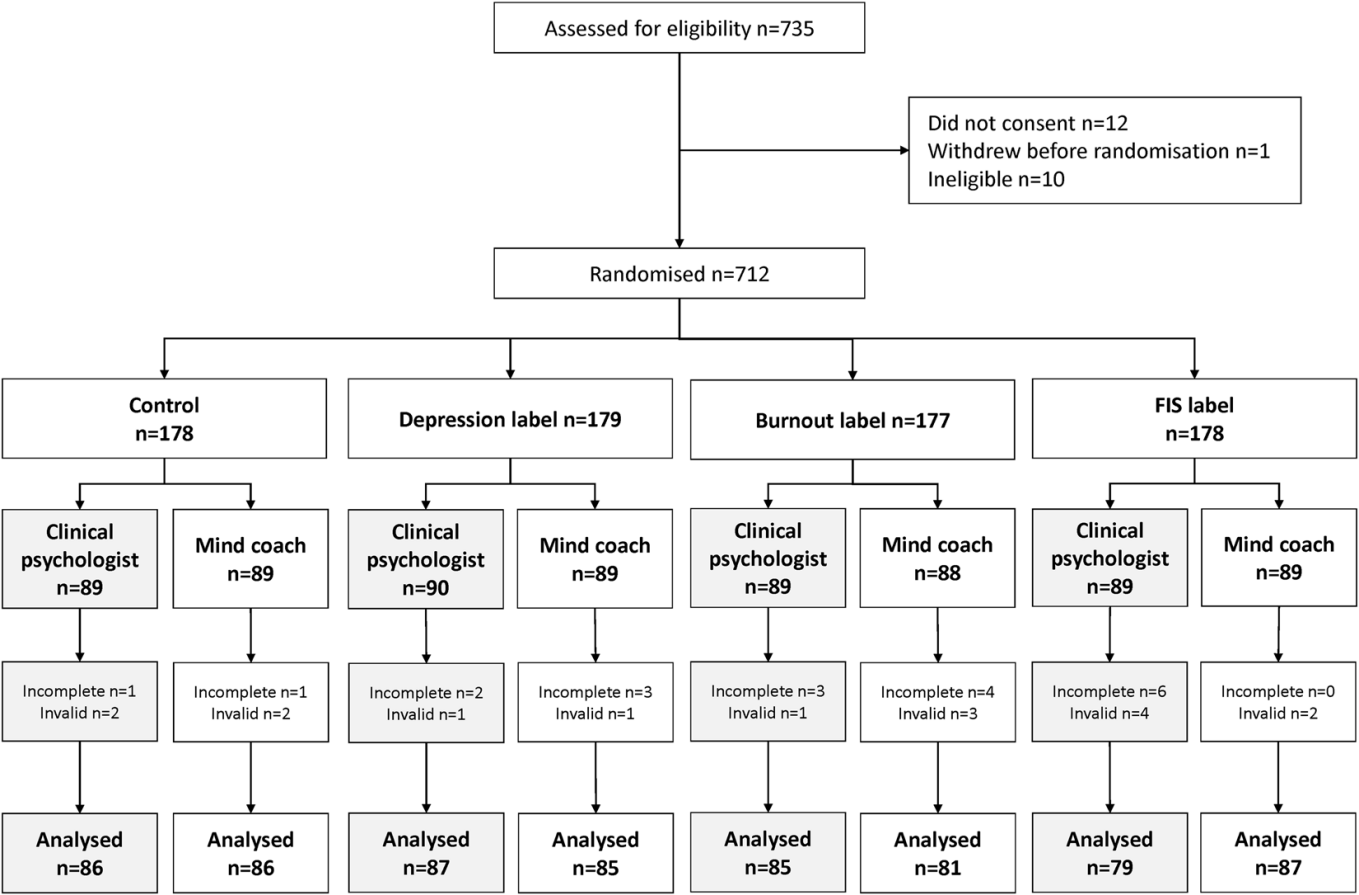
. Participant flow diagram. FIS, functional impairment syndrome.

Of the 735 participants who were recruited and assessed for eligibility between 13th and 14th January 2021, 12 did not consent to participation, 10 were ineligible, 1 withdrew before randomization, 20 withdrew after randomization and 16 responses were deemed invalid due to short completion time (Figure 1). This left 676 participant responses for analysis.

Socio‐demographic and health characteristics by diagnostic label and overall are shown in Table 2. Unadjusted means and standard deviations by diagnostic label are shown in Table 3. Unadjusted means and standard deviations by recommendation label are shown in Table 4.

**Table 2:** Demographic characteristics by diagnostic label group

Factor	Level	Control (*n* = 172)	Depression (*n* = 172)	Burnout (*n* = 166)	Functional impairment syndrome (*n* = 166)	Overall (*n* = 676)
Gender (What is the gender you identify as?)	Female	91 (52.9%)	89 (51.7%)	86 (51.8%)	90 (54.2%)	356 (52.7%)
	Male	81 (47.1%)	82 (47.7%)	80 (48.2%)	76 (45.8%)	319 (47.2%)
	Another identity^a^	0 (0.0%)	1 (0.6%)	0 (0.0%)	0 (0.0%)	1 (0.1%)
Age, mean (SD)		43.26 (13.71)	41.45 (13.80)	42.31 (14.26)	42.28 (13.94)	42.33 (13.91)
Highest level of education^b^	University degree	68 (39.5%)	74 (43.0%)	78 (47.0%)	78 (47.0%)	298 (44.1%)
	Diploma, certificate or trade apprenticeship	51 (29.6%)	42 (24.4%)	33 (19.9%)	51 (30.7%)	177 (26.2%)
	Higher school certificate or leaving certificate (or equivalent)	35 (20.3%)	39 (22.7%)	33 (19.9%)	24 (14.5%)	131 (19.4%)
	School certificate or intermediate certificate (or equivalent)	15 (8.7%)	13 (7.6%)	19 (11.4%)	11 (6.6%)	58 (8.6%)
	No school or other qualifications	3 (1.7%)	4 (2.3%)	3 (1.8%)	2 (1.2%)	12 (1.8%)
Country of birth	Australia	134 (77.9%)	136 (79.1%)	124 (74.7%)	133 (80.1%)	527 (78.0%)
	New Zealand, UK, or Europe	18 (10.4%)	18 (10.5%)	16 (9.6%)	9 (5.4%)	61 (9.1%)
	Asia	17 (9.9%)	15 (8.7%)	23 (13.9%)	22 (13.3%)	77 (11.4%)
	Other	3 (1.7%)	3 (1.7%)	3 (1.8%)	2 (1.2%)	11 (1.6%)
Relationship status	Single	49 (28.5%)	50 (29.1%)	52 (31.3%)	48 (28.9%)	199 (29.4%)
	Dating	7 (4.1%)	7 (4.1%)	8 (4.8%)	11 (6.6%)	33 (4.9%)
	Married or defacto/living with partner	107 (62.2%)	104 (60.4%)	96 (57.9%)	100 (60.2%)	407 (60.2%)
	Separated/divorced or widowed	9 (5.2%)	11 (6.4%)	10 (6.0%)	7 (4.2%)	37 (5.5%)
Clinically diagnosed Depression and/or Anxiety	Neither	113 (65.7%)	99 (57.6%)	115 (69.3%)	100 (60.2%)	427 (63.2%)
	Depression only	14 (8.1%)	22 (12.8%)	15 (9.0%)	23 (13.9%)	74 (10.9%)
	Anxiety only	20 (11.6%)	26 (15.1%)	9 (5.4%)	16 (9.6%)	71 (10.5%)
	Both	25 (14.5%)	25 (14.5%)	27 (16.3%)	27 (16.3%)	104 (15.4%)
Current employment status	Full time employment	85 (49.4%)	88 (51.2%)	68 (41.0%)	75 (45.2%)	316 (46.7%)
	Part time employment	26 (15.1%)	39 (22.7%)	34 (20.5%)	39 (23.5%)	138 (20.4%)
	Studying	7 (4.1%)	8 (4.7%)	13 (7.8%)	11 (6.6%)	39 (5.8%)
	Home‐duties	20 (11.6%)	14 (8.1%)	16 (9.6%)	21 (12.7%)	71 (10.5%)
	Retired	17 (9.9%)	12 (7.0%)	22 (13.3%)	12 (7.2%)	63 (9.3%)
	Other	17 (9.9%)	11 (6.4%)	13 (7.8%)	8 (4.8%)	49 (7.2%)
Current self‐report depression symptoms (PHQ‐9) total score, median (IQR) (range 0–27)		4 (1, 10)	6 (1, 12)	5 (1, 12)	5 (1, 12)	5 (1, 12)

*Note*: Data are displayed as frequency (n) and relative frequency (%) unless otherwise specified. All median scores are below accepted cut‐offs for detecting depression (Manea et al., 2011). Abbreviations: FIS, functional impairment syndrome; PHQ: patient health questionnaire‐9.

^a^ The one participant who reported another gender identity received the female version of the scenario

^b^ <2% reported no qualifications.

### 3.1 Primary outcome

#### 3.1.1 Help‐seeking intention (scale 1–5; higher = higher intention)

There was no evidence of an overall effect of diagnostic label on help‐seeking intention (*F *[3, 667] = 1.86, *p* = 0.13). There was very strong statistical evidence of an overall effect of recommendation on help‐seeking intention (*F *[1, 667] = 22.99, *p* < 0.001). Intention to seek help was higher for the clinical psychologist (*M* = 3.77, 95% CI: 3.64–3.89) than mind coach recommendation (*M* = 3.34, 95% CI: 3.21–3.46; MD = 0.43, 95% CI: 0.25–0.60; *p* < 0.001). There was no statistical evidence for a diagnostic label‐by‐recommendation label interaction ([3, 667] = 1.74, *p* = 0.16).

**Table 3:** Outcomes by randomized diagnostic label groups

Outcome	Control (*n* = 172)	Depression (*n* = 172)	Burnout (*n* = 166)	FIS (*n* = 166)	Main effect *p*‐value
Intention to seek help (1–5)	3.58 (1.20)	3.69 (1.12)	3.40 (1.20)	3.52 (1.19)	0.13
Intention to speak with boss (1–5)	3.15 (1.22)	3.31 (1.23)	2.91 (1.28)	3.13 (1.20)	0.027
Self‐stigma (16–80)^a^	49.20 (12.81)	49.76 (12.20)	49.86 (11.54)	52.06 (11.89)	0.13
Worry (1–7)	4.98 (1.34)	5.00 (1.45)	4.86 (1.48)	4.88 (1.39)	0.73
Perceived severity (1–7)	5.06 (1.23)	5.54 (1.10)	5.08 (1.20)	4.89 (1.38)	<0.001
Brief‐IPQ (0–10)					
*Consequences*	7.04 (2.25)	7.30 (2.07)	6.88 (2.35)	6.72 (2.29)	0.094
*Timeline*	5.42 (2.38)	5.82 (2.25)	5.31 (2.37)	5.45 (2.37)	0.21
*Personal control*	5.72 (2.33)	5.54 (2.41)	5.43 (2.46)	5.60 (2.35)	0.72
*Treatment control*	6.06 (2.84)	6.51 (2.51)	5.86 (2.81)	6.18 (2.66)	0.15
Personal stigma (0–36)	14.92 (8.21)	13.77 (9.06)	15.34 (7.59)	15.82 (8.05)	0.098

*Note*: Data are shown as unadjusted means (standard deviation).

^a^ Mean selfstigma scores by severity level of depression: no depression = 57.76, mild depression = 59.04, moderate to severe depression = 61.05.

**Table 4:** Outcomes by randomized recommendation label groups.

Outcome	Clinical psychologist (*n* = 337)	Mind coach (*n* = 339)	Main effect *p*‐value
Intention to seek help (1–5)	3.77 (1.05)	3.34 (1.26)	<0.001
Intention to speak with boss (1–5)	3.14 (1.20)	3.11 (1.28)	0.73
Self‐stigma (16–80)	49.28 (11.75)	51.12 (12.49)	0.060
Worry (1–7)	4.91 (1.40)	4.94 (1.43)	0.78
Perceived severity (1–7)	5.15 (1.21)	5.14 (1.29)	0.93
Brief‐IPQ (0–10)			
*Consequences*	6.93 (2.14)	7.05 (2.35)	0.45
*Timeline*	5.54 (2.22)	5.47 (2.46)	0.65
*Personal control*	5.55 (2.29)	5.60 (2.48)	0.82
*Treatment control*	6.50 (2.50)	5.81 (2.87)	<0.001
Personal stigma (0–36)	14.84 (7.96)	15.06 (8.58)	0.78

*Note*: Data are shown as unadjusted means (standard deviation).

### 3.2 Secondary outcomes

#### 3.2.1 Intention to speak with boss (scale 1–5; higher = higher intention)

There was evidence of a main effect of diagnostic label for intention to speak to boss (*F *[3, 667] = 3.08, *p* = 0.027), but no effect of recommendation label (*F *[1, 667] = 0.12, *p* = 0.73) or diagnostic label‐by‐recommendation label interaction (*F *[3, 667] = 0.52, *p* = 0.67). Pairwise comparisons suggested higher intentions to speak to a boss with the depression label (*M* = 3.31, 95% CI: 3.12–3.49) compared to the burnout label (*M* = 2.90, 95% CI: 2.72–3.09; MD = 0.40, 95% CI: 0.14–0.66; *p* = 0.003). No other pairwise comparisons showed evidence of statistical significance.

#### 3.2.2 Self‐stigma (score range 16–80; higher = higher stigma)

There was no evidence of an overall effect of diagnostic label on self‐stigma (*F *[3, 667] = 1.86, *p* = 0.13). There was also no evidence of a main effect of recommendation label (*F *[1, 667] = 3.55, *p* = 0.060), and no diagnostic label‐by‐recommendation label interaction (*F *[3, 667] = 1.46, *p* = 0.22).

#### 3.2.3 Worry (scale 1–7; higher = greater worry)

There was no evidence of a main effect of diagnostic label (*F *[3, 667] = 0.43, *p* = 0.73), no effect of recommendation label (*F *[1, 667] = 0.08, *p* = 0.78), and no diagnostic label‐by‐recommendation label interaction (*F *[3, 667] = 0.17, *p* = 0.92) for worry.

#### 3.2.4 Perceived severity (scale 1–7, higher = more severe)

There was very strong evidence of a main effect of diagnostic label for perceived severity (*F *[3, 667] = 8.55, *p* < 0.001). Pairwise comparisons suggested higher perceived severity for the depression label (*M* = 5.54, 95% CI: 5.36–5.73) compared to all other groups (control: *M* = 5.06, 95% CI: 4.87–5.24; MD = 0.48, 95% CI: 0.22–0.74; *p* < 0.001; burnout: *M* = 5.08, 95% CI: 4.89, 5.27; MD = 0.46, 95% CI: 0.20–0.73; *p* = 0.001; FIS: *M* = 4.90, 95% CI: 4.71–5.09; MD = 0.64, 95% CI: 0.38–0.91; *p* < 0.001). There was no effect of recommendation label (*F *[1, 667] = 0.01, *p* = 0.93) or diagnostic label‐by‐recommendation label interaction (*F *[3, 667] = 1.52, *p* = 0.21).

#### 3.2.5 Cognitive representation (Brief‐IPQ; scale 0–10; higher = more negative perceptions)

##### IPQ—consequences

There was no evidence of a main effect of diagnosis (*F *[3, 667] = 2.14, *p* = 0.094), no effect of recommendation label (*F *[1, 667] = 0.57, *p* = 0.45), and no diagnostic label‐by‐recommendation label interaction (*F *[3, 667] = 0.63, *p* = 0.60) for IPQ‐Consequence. IPQ‐Consequences was slightly lower for the FIS label (*M* = 6.71, 95% CI: 6.37–7.06) than depression label (*M* = 7.31, 95% CI: 6.97–7.64, MD = 0.59, 95% CI: 0.11–1.07; *p* = 0.015).

##### IPQ—timeline

There was no evidence of a main effect of diagnostic label (*F *[3, 667] = 1.53, *p* = 0.21), no effect of recommendation label (*F *[1, 667] = 0.21, *p* = 0.65), and no diagnostic label‐by‐recommendation label interaction (*F *[3, 667] = 2.23, *p* = 0.08) for IPQ‐Timeline.

##### IPQ—personal control

There was no evidence of a main effect of diagnostic label (*F *[3, 667] = 0.45, *p* = 0.72), no effect of recommendation label (*F *[1, 667] = 0.05, *p* = 0.82), and no diagnostic label‐by‐recommendation label interaction (*F *[3, 667] = 0.54, *p* = 0.66) for IPQ‐personal control.

##### IPQ—treatment control

There was no evidence of a main effect of diagnostic label (*F *[3, 667] = 1.79, *p* = 0.15) for IPQ‐treatment control. Treatment control was slightly lower for the burnout label (*M* = 5.84, 95% CI: 5.43–6.25) than depression label (*M* = 6.50, 95% CI: 6.10–6.90; MD = 0.66, 95% CI: 0.09–1.23; *p* = 0.024). There was very strong evidence of a main effect of recommendation label for IPQ‐Treatment Control (*F *[1, 667] = 11.07, *p* < 0.001). Treatment control was greater with the clinical psychologist recommendation (*M* = 6.50, 95% CI: 6.21–6.78) than for the mind coach recommendation (*M* = 5.81, 95% CI: 5.52–6.09; MD = 0.69, 95% CI: 0.29–1.10; *p* = 0.001). There was no diagnostic label‐by‐recommendation label interaction (*F *[3, 667] = 2.23, *p* = 0.08).

#### 3.2.6 Personal stigma (score range 0–36; higher = greater stigma)

There was no evidence of a main effect of diagnostic label (*F *[3, 667] = 2.11, *p* = 0.098), no effect of recommendation label (*F *[1, 667] = 0.08, *p* = 0.78), and no diagnostic label‐by‐recommendation label interaction (*F *[3, 667] = 1.12, *p* = 0.34) for personal stigma. Personal stigma was slightly higher for the FIS (*M* = 15.85, 95% CI: 14.63–17.08) than the depression label (*M* = 13.74, 95% CI: 12.54–14.94; MD = 2.11, 95% CI: 0.39–3.83; *p* = 0.016).

### 3.3 Exploratory analysis

No significant interactions were found for any individual factors by diagnostic label or by recommendation label. See Supporting Information for detailed results.

## 4 DISCUSSION

In our study, participants who received the depression label reported higher intention to speak with their boss compared to those who received the burnout label, and greater perceived seriousness of the condition compared to any other diagnostic label. Although we found no differences in help‐seeking intentions across diagnostic labels, participants who received the clinical psychologist recommendation reported higher intention to seek help and greater perceived helpfulness of treatment compared to those who received the mind coach recommendation.

In contrast to previous research suggesting a burnout diagnosis is associated with lower self‐stigma than depression (Bianchi et al., 2016), we found no differences in stigma between these diagnostic labels. Furthermore, we found that participants who received the depression diagnosis reported higher intention to speak to their boss about what their GP had told them compared to those who received the burnout diagnosis. Although there may be concern regarding the stigma surrounding depression and clinical psychologists, our results suggest that providing a burnout diagnosis to explain mild depressive symptoms in workplace/occupational contexts may not be more favorable in terms of alleviating stigma and increasing help‐seeking. Given greater efforts to reduce stigma surrounding depression through awareness raising, this may currently be a preferred diagnostic label to ensure that those experiencing such symptoms feel that their condition is valid. This could be because people have greater appreciation of the dimensional nature of depression and the label is now less likely to invoke stereotypes (Corrigan, 2007), in addition to greater education about “recovery‐oriented” models of care in mental health that challenge the notion of people with mental illness failing to respond to treatment (Corrigan, 2007). Our findings may have differed to Bianchi and colleagues' findings of reduced stigma with the burnout label (Bianchi et al., 2016) for this reason. Attitudes toward depression may have improved since this study was conducted in 2015. Furthermore, Bianchi and colleagues' sample included teachers only, and the study design was not randomized (Bianchi et al., 2016). The effect of reduced stigma as a result of the burnout label may have been due to a factor other than the labels and may not have the same impact in more heterogeneous populations that are less industry specific or when a randomized design is used. Findings from the World Health Organization's Mental Health Survey also found that attitudinal barriers, such as self‐ and perceived‐stigma, are more salient for people with more severe mental health conditions (Andrade et al., 2014). However, our study scenarios described mild to moderate depressive symptoms, meaning these barriers may not be as relevant. Furthermore, low‐perceived need is one of the strongest deterrents from mental health help‐seeking (Andrade et al., 2014). In our study, participants may have perceived a greater need for treatment for depression given the higher perceived severity reported with this label compared to all other groups.

There is currently much debate surrounding burnout and distinguishing it from the diagnostic category of depression. It is not yet certain whether they can be treated as distinct diagnostic entities due to the significant overlap in symptoms when considered in its current conceptualization (Bianchi et al., 2015). However, recent research in Australia has begun to re‐examine what constructs best represent burnout, how it can be measured distinctly from depression, and the personality types most prone to burnout (Tavella & Parker, 2020). If burnout is eventually considered a distinct diagnostic entity, further efforts will likely be needed to educate and inform the community about the similarities and differences between the conditions. There may be a need to focus on perceptions of burnout as a valid diagnosis that may require psychological treatment for alleviation of symptoms and reduced workload or time off work.

Our findings also suggest that being advised to see a clinical psychologist may instil more confidence in those experiencing mild symptoms of depression compared to a recommendation to see a mind coach. This has implications for individuals and organizations using alternative terminology for help‐seeking. For example, Beyond Blue, a leading Australian mental health organization has recently developed “NewAccess,” a free mental health coaching program, whereby Australians can access low‐intensity Cognitive Behavioral Therapy through guidance from a local coach (Beyond Blue, 2021). Another example of the use of alternative terminology is “MindSpot,” a free service that provides assessment and treatment courses online (including weekly contact from experienced therapists) for Australian adults who are experiencing difficulties with symptoms of mental ill health (MindSpot, 2021). When using terminology that differs to what a lay community individual might expect, additional efforts may be required to ensure those who require the service can have confidence in its ability to improve their symptoms. It may be helpful, for example, to specifically acknowledge how exactly the alternative may be similar and/or different to a clinical psychologist (who they have come to expect to be the necessary and effective treatment). Importantly, we did not assess the difference between receiving a recommendation to see a clinical psychologist with a true control recommendation such as no treatment, watchful waiting or taking time off work. Future research should examine the impact of a broader range of treatment recommendations to especially understand how they differ to recommendations for no or delayed treatment (e.g., a discrete choice experiment with vignettes could explore choices for different treatment options and factors influencing the choice).

In other medical contexts where disease labels have been investigated, evidence of increased perceived severity can be interpreted as an adverse impact of labeling. For example, when examining the impacts of a polycystic ovary syndrome diagnosis, increased perceived severity may be a negative outcome due to the greater likelihood of choosing medical interventions that could lack benefit for women experiencing milder symptoms that may be alleviated through healthy lifestyle changes (Copp et al., 2017). Although increased perceived severity with the depression diagnosis may be interpretated as a positive outcome due to the success of educational efforts surrounding its seriousness, it is also important to acknowledge the potential for negative impacts. There may be a particular need for caution when communicating a diagnosis of depression to people with mild symptoms which may spontaneously remit without any specific intervention, or whose symptoms are a “normal” response to a stressful situation (Parker, 2007). An increase in perceived seriousness of the condition in this case may not necessarily translate to greater help‐seeking or may result in treatment that is unlikely to be beneficial, so providing a diagnosis that may not benefit the patient and could instead have negative psychological impacts. Given that we found no other differences in outcomes across the depression label and control groups, further research would be useful to understand any positive or negative impacts of receiving the depression label when experiencing milder depressive symptoms. In particular, qualitative research may be useful to identify impacts that cannot be pre‐empted and assessed in quantitative, experimental research. Given the label‐related differences identified in Bianchi and colleagues' study^2016^, future research could also explore how individuals vary in their responses to the depression label depending on characteristics such as age, gender, occupation, as our study was not powered to detect such differences.

There are several limitations to this study that should be acknowledged. Firstly, our design used a hypothetical scenario and a community sample for which the content of the scenario may be more or less realistic for certain individuals. However, our randomized design accounts for any potential differences and comments provided by participants in free‐text responses indicated they were engaged in the content of the scenario (as there were very few blank responses, and participants often referred to the scenario or provided insight into the thinking behind their intention rating). Secondly, our findings should be interpreted in light of the COVID‐19 pandemic as public discussion about mental health and help‐seeking has substantially increased, with Australian data showing a recent overall increase in mental health presentations during COVID‐19 for depression and anxiety (Academic Unit of General Practice, 2021). Greater access to government funding for clinical psychologists has also been made available and collectively these changes may have contributed to increased help‐seeking for depression. Results may have differed if this study were conducted before the pandemic. Recent international data has shown that it may in fact be social norms that influence mental health help‐seeking during COVID‐19 rather than selfstigma, (Lueck, 2021) but this factor was not assessed in the design of this study. Our study was also limited by not including a control condition to compare the recommendation labels against, meaning we could not compare the impact of terminology for treatments to a no treatment alternative. We also acknowledge that information about the gender identification of participants, but not biological sex was reported, which future research should ensure to ascertain, as differences in responses may have biological underpinnings. Finally, the primary outcome assessed was intention, not actual behavior.

## 5 CONCLUSION

Findings from this study highlight how providing a depression diagnosis for mild depressive symptoms may result in greater perceived severity of the condition without necessarily translating to greater help‐seeking intention. We also found that referral to a mental healthcare provider described as a “clinical psychologist” rather than a “mind coach” may result in greater intention to seek help and provide a greater sense of treatment control. Our results do not support previous findings that a diagnosis of burnout would be less stigmatizing than depression. Moreover, we found that people diagnosed with burnout could be less likely to discuss it with their employer than people diagnosed with depression. Future research is needed to understand how individuals may vary in response to different terminology for depressive symptoms and treatment recommendations in other contexts (including experiences of more severe symptoms). The differences evident across labeling conditions in our study further highlight the importance and complexity of labeling impacts in the mental health context, including the potential considerations for future research to explore impacts of terminology in other settings where public attitudes may be even more negative (e.g., schizophrenia and bipolar disorder) (Wood et al., 2014).

## CONFLICT OF INTEREST

The authors declare no conflict of interest.

### PEER REVIEW

The peer review history for this article is available at https://publons.com/publon/10.1002/jclp.23410

## ETHICS STATEMENT

The study was approved by the University of Sydney Human Research Ethics Committee (2020/058).

## Supporting information

Supplementary information.

## Data Availability

The data that support the findings of this study are available from the corresponding author upon reasonable request.
