# Supplementary material for: Impact of alternative terminology for depression on help‐seeking intention: A randomized online trial
Source: J Clin Psychol. 2022 Jul 8;79(1):68–85. doi: 10.1002/jclp.23410 (PMC10953448; doi:10.1002/jclp.23410)
Supplement: Supplementary file 1 — Supplementary information. [file JCLP-79-68-s001.pdf]

SUPPLEMENTARY MATERIAL

Labels and recommendations given in hypothetical scenario

**Group 1: No label**<br>Your general practitioner considers what you have said, asks you to complete a questionnaire and says…<br>*"It sounds like what you are feeling is leading to difficulties at work, and some issues with your home life and your relationships."*	
**Group 1A:**<br>*"What I suggest is seeing a* ***clinical psychologist*** *qualified in evidence-based strategies to help you feel better about things. I could refer you if you would like?"*	**Group 1B:**<br>*"What I suggest is seeing a* ***mind coach*** *qualified in evidence-based strategies to help you feel better about things. I could refer you if you would like?"*

**Group 2: Depression label**<br>Your general practitioner considers what you have said, asks you to complete a questionnaire and says…<br>*"I think you have* ***depression,*** *because it sounds like what you are feeling is leading to difficulties at work, and some issues with your home life and your relationships."*	
**Group 2A:**<br>*"What I suggest is seeing a* ***clinical psychologist*** *qualified in evidence-based strategies to help you feel better about things. I could refer you if you would like?"*	**Group 2B:**<br>*"What I suggest is seeing a* ***mind coach*** *qualified in evidence-based strategies to help you feel better about things. I could refer you if you would like?"*

**Group 3: Burnout label**<br>Your general practitioner considers what you have said, asks you to complete a questionnaire and says…<br>*"I think you have* ***burnout,*** *because it sounds like what you are feeling is leading to difficulties at work, and some issues with your home life and your relationships."*	
**Group 3A:**<br>*"What I suggest is seeing a* ***clinical psychologist*** *qualified in evidence-based strategies to help you feel better about things. I could refer you if you would like?"*	**Group 3B:**<br>*"What I suggest is seeing a* ***mind coach*** *qualified in evidence-based strategies to help you feel better about things. I could refer you if you would like?"*

**Group 4: Functional impairment syndrome label**<br>Your general practitioner considers what you have said, asks you to complete a questionnaire and says…<br>*"I think you have* ***functional impairment syndrome,*** *because it sounds like what you are feeling is leading to difficulties at work, and some issues with your home life and your relationships."*	
**Group 4A:**<br>*"What I suggest is seeing a* ***clinical psychologist*** *qualified in evidence-based strategies to help you feel better about things. I could refer you if you would like?"*	**Group 4B:**<br>*"What I suggest is seeing a* ***mind coach*** *qualified in evidence-based strategies to help you feel better about things. I could refer you if you would like?"*

Exploratory analyses

Analysis by gender (scenario received):

Individuals who received the male scenario had greater intention to speak with their boss, and higher IPQ-personal control, but also higher personal stigma and IPQ-timeline compared to those who received the female scenario. There was no evidence of interactions of scenario received with either label or recommendation.

Estimated marginal means (95%CIs)

Outcome	Female	Male	p-value
Intention to seek help (1-5)	3.49 (3.37, 3.61)	3.62 (3.49, 3.74)	.17
Intention to speak with boss (1-5)	2.96 (2.83, 3.08)	3.31 (3.18, 3.45)	<.001
Self-stigma (16-80)	49.88 (48.62, 51.14)	50.57 (49.24, 51.90)	.46
Worry (1-7)	4.95 (4.80, 5.09)	4.91 (4.75, 5.07)	.73
Perceived severity (1-7)	5.11 (4.98, 5.24)	5.19 (5.05, 5.32)	.40
Brief-IPQ (0-10)			
Consequences	7.02 (6.79, 7.26)	6.95 (6.70, 7.20)	.67
Timeline	5.32 (5.08, 5.57)	5.70 (5.45, 5.96)	.034
Personal Control	5.21 (4.97, 5.46)	5.97 (5.71, 6.23)	<.001
Treatment Control	5.99 (5.71, 6.27)	6.33 (6.04, 6.63)	.095
Personal stigma (0-36)	13.18 (12.34, 14.01)	16.94 (16.06, 17.83)	<.001

Explore interactions of sex_scenario by label and sex_scenario by recommendation

Outcome	Scenario-by-Label	Scenario-by-Recommendation
Intention to seek help (1-5)	.82	.28
Intention to speak with boss (1-5)	.99	.66
Self-stigma (16-80)	.38	.96
Worry (1-7)	.84	.26
Perceived severity (1-7)	.91	.44
Brief-IPQ (0-10)		
Consequences	.99	.24
Timeline	.44	.88
Personal Control	.39	.92
Treatment Control	.75	.59
Personal stigma (0-36)	.41	.62

Analysis by education (university vs not):

Added education into models (university vs less than university). Higher education is associated with higher values on all outcomes (except treatment control), but there is no convincing evidence of interaction with label or recommendation

Outcome	Less than university	University	p-value
Intention to seek help (1-5)	3.43 (3.32, 3.55)	3.70 (3.57, 3.83)	.003
Intention to speak with boss (1-5)	3.01 (2.89, 3.14)	3.27 (3.13, 3.41)	.007
Self-stigma (16-80)	49.30 (48.08, 50.52)	51.35 (49.97, 52.72)	.03
Worry (1-7)	4.78 (4.64, 4.92)	5.12 (4.96, 5.28)	.002
Perceived severity (1-7)	5.01 (4.89, 5.14)	5.31 (5.17, 5.45)	.002
Brief-IPQ (0-10)			
Consequences	6.70 (6.48, 6.93)	7.35 (7.10, 7.61)	<.001
Timeline	5.29 (5.05, 5.52)	5.77 (5.51, 6.04)	.007
Personal Control	5.26 (5.02, 5.50)	5.97 (5.70, 6.23)	<.001
Treatment Control	5.98 (5.71, 6.25)	6.37 (6.06, 6.67)	.066
Personal stigma (0-36)	13.59 (12.79, 14.39)	16.68 (15.78, 17.58)	<.001

Explore interactions of education by label and education by recommendation

Outcome	Education-by-Label	Education-by-Recommendation
Intention to seek help (1-5)	.26	.049
Intention to speak with boss (1-5)	.42	.15
Self-stigma (16-80)	.35	.91
Worry (1-7)	.35	.21
Perceived severity (1-7)	.53	.20
Brief-IPQ (0-10)		
Consequences	.92	.21
Timeline	.32	.49
Personal Control	.13	.51
Treatment Control	.63	.51
Personal stigma (0-36)	.31	.95

Analysis by mental health history (none vs dep, anx, or both)

Added history of mental health into models (none vs any (anxiety, depression, or both). See below for differences; no convincingly strong evidence of effect modification.

Outcome	None	Any (Dep, Anx, or Both)	p-value
Intention to seek help (1-5)	3.45 (3.34, 3.56)	3.73 (3.59, 3.87)	.002
Intention to speak with boss (1-5)	3.05 (2.94, 3.17)	3.25 (3.09, 3.40)	.052
Self-stigma (16-80)	49.38 (48.23, 50.53)	51.61 (50.10, 53.12)	.022
Worry (1-7)	4.69 (4.56, 4.82)	5.34 (5.17, 5.51)	<.001
Perceived severity (1-7)	4.97 (4.86, 5.09)	5.44 (5.29, 5.59)	<.001
Brief-IPQ (0-10)			
Consequences	6.55 (6.34, 6.76)	7.74 (7.46, 8.00)	<.001
Timeline	5.08 (4.86, 5.30)	6.23 (5.95, 6.51)	<.001
Personal Control	5.56 (5.34, 5.79)	5.58 (5.29, 5.88)	.92
Treatment Control	5.85 (5.59, 6.10)	6.68 (6.35, 7.01)	<.001
Personal stigma (0-36)	15.00 (14.23, 15.77)	14.88 (13.67, 15.88)	.85

Explore interactions of mental health history by label and mental health history by recommendation

Outcome	MH Hx-by-Label	MH Hx-by-Recommendation
Intention to seek help (1-5)	.048	.98
Intention to speak with boss (1-5)	.82	.34
Self-stigma (16-80)	.33	.57
Worry (1-7)	.08	.65
Perceived severity (1-7)	.07	.72
Brief-IPQ (0-10)		
Consequences	.43	.79
Timeline	.71	.034
Personal Control	.69	.34
Treatment Control	.10	.86
Personal stigma (0-36)	.46	.88

Analysis by depression history (none or anx only vs dep only or both)

Added history of depression into models (none or anxiety only, vs depression only or both anxiety and depression). See below for differences; no evidence of effect modification.

Outcome	None or Anxiety only	Depression or Both	p-value
Intention to seek help (1-5)	3.45 (3.35, 3.55)	3.84 (3.67, 4.01)	<.001
Intention to speak with boss (1-5)	3.07 (2.96, 3.17)	3.29 (3.11, 3.47)	.035
Self-stigma (16-80)	49.99 (48.92, 51.06)	50.80 (49.00, 52.59)	.45
Worry (1-7)	4.75 (4.63, 4.87)	5.43 (5.23, 5.64)	<.001
Perceived severity (1-7)	5.00 (4.90, 5.11)	5.54 (5.36, 5.72)	<.001
Brief-IPQ (0-10)			
Consequences	6.63 (6.44, 6.82)	7.98 (7.66, 8.30)	<.001
Timeline	5.22 (5.02, 5.42)	6.30 (5.96, 6.64)	<.001
Personal Control	5.60 (5.39, 5.80)	5.50 (5.15, 5.85)	.65
Treatment Control	5.92 (5.68, 6.15)	6.81 (6.42, 7.21)	<.001
Personal stigma (0-36)	15.32 (14.61, 16.03)	13.92 (12.73, 15.11)	.049

Explore interactions of mental health history by label and mental health history by recommendation

Outcome	Depression Hx-by-Label	Depression Hx-by-Recommendation
Intention to seek help (1-5)	.28	.22
Intention to speak with boss (1-5)	.63	.06
Self-stigma (16-80)	.11	.44
Worry (1-7)	.23	.94
Perceived severity (1-7)	.31	.32
Brief-IPQ (0-10)		
Consequences	.64	.66
Timeline	.57	.06
Personal Control	.32	.62
Treatment Control	.47	.30
Personal stigma (0-36)	.35	.73
